# Chest physiotherapy improves regional lung volume in ventilated children

**DOI:** 10.1186/s13054-020-03156-2

**Published:** 2020-07-16

**Authors:** Bronagh McAlinden, Suzanne Kuys, Andreas Schibler, Judith L. Hough

**Affiliations:** 1 School of Allied Health, Australian Catholic University, Banyo, QLD 4014 Australia; 2 Mater Health, South Brisbane, 4101 Australia; 3 Paediatric Critical Care Research Group, Children’s Health Research Centre – The University of Queensland, South Brisbane, 4101 Australia

**Keywords:** Electrical impedance tomography, Chest physiotherapy, Ventilation distribution, Paediatric, Suction

Dear Editor,

Chest physiotherapy (CPT) is widely used to improve distribution of ventilation and gas exchange in the management of mechanically ventilated infants and children with lung disease [1]. The mechanism by which CPT works is not well understood due to the lack of appropriate outcome measures capable of quantifying changes in ventilation distribution. Electrical impedance tomography (EIT), a non-invasive means of measuring ventilation distribution, is a potential tool to measure CPT effects on lung function in ventilated infants and children [2]. We describe, using EIT, the effect of CPT compared with receiving endotracheal suction only on ventilation distribution and gas exchange in children.

A secondary analysis of data previously collected within a prospective randomised controlled trial investigating the effect of recruitment manoeuvres on 60 ventilated children following endotracheal (ETT) suction was conducted in a tertiary paediatric intensive care unit [3]. Children who, based on clinical indication, had received CPT for intensive airway clearance were compared to children receiving suction only. CPT compromised any combination of manual techniques and manual hyperinflation followed by open ETT suctioning [1].

Ventilation distribution (amplitude, global ventilation inhomogeneity, geometric centre) and end-expiratory lung volume (EELV) were measured using EIT (Gottingen GoeMF II, VIASYS Healthcare, Hochberg, Germany) prior to CPT and suction, and then 30, 60 and 120 min post-intervention. Gas exchange (arterial blood gases and oxygen saturation) and physiological variables (heart rate and respiratory rate) were recorded.

Linear mixed models were used to determine differences and interactions between those who did and did not receive CPT, over the four time points, for each dependent variable. As this was a secondary analysis of data, we also examined interactions with recruitment manoeuvres and found that the effects of CPT were independent of lung recruitment manoeuvres (*p* > 0.05).

Seventeen participants (28%) received CPT (28.7 ± 49.3 months), and forty-three participants (72%) received no CPT (47.8 ± 55.8 months) (*p* = 0.22). Ventilator settings remained constant pre- and post-intervention. No differences were found at baseline between the two groups for all parameters except PaCO_2_, which was significantly higher in the CPT group (Table 1), indicative of ventilation maldistribution, more extensive lung disease and was a clinical trigger for CPT. Similar to previous studies, the differences we found in CO_2_ between CPT and suction remained consistent after intervention [4, 5].

**Table 1:** Participant characteristics for the chest physiotherapy (CPT) group and routine airway clearance group at baseline: mean (SD)

Characteristic	CPT (*n* = 17)	Routine airway clearance (*n* = 43)	Sig (*p* value)
Age (months)	28.7 (49.3)	47.8 (55.8)	0.221
Weight (kg)	11.1 (11.4)	16.6 (14.2)	0.163
ETT size (mm)	4.2 (1.1)	4.5 (1.2)	0.328
Baseline FiO_2_	0.4 (0.1)	0.4 (0.1)	0.834
Baseline PaO_2_ (mmHg)	89.4 (30.2)	96.4 (29.6)	0.411
Baseline PaCO_2 (_mmHg)	58.7 (11.1)	48.6 (11.5)	*0.003
Baseline P/F ratio	282.3 (165.9)	286.9 (109.6)	0.901
Baseline RR (breaths/min)	34.7 (17.5)	28.7 (11.2)	0.121
Baseline PEEP (cmH_2_O)	7.0 (2.4)	7.8 (2.2)	0.239
Baseline PIP (cmH_2_O)	21.1 (7.0)	21.2 (4.3)	0.932
Baseline MAP (cmH_2_O)	11.0 (3.6)	11.0 (3.0)	0.939
Cuffed ETT, *n* (%)	14 (92%)	38 (88%)	0.834
**Ventilation mode, number (%)**			
SIMV	16 (94%)	39 (91%)	0.424
PCV	0	1 (2.3%)	–
PSV	1 (6%)	2 (4.7%)	–
CPAP	0	1 (2.3%)	–
**Randomised groups, number (%)**			
Control	7 (41%)	13 (30%)	
Double PEEP recruitment	4 (24%)	16 (37%)	
Stepwise recruitment	6 (35%)	14 (33%)	
**Reason for intubation, number (%)**			
Primary respiratory pathology#	10 (59%)	21 (49%)	0.226
Secondary respiratory pathology^	7 (41%)	22 (51%)	–

*Abbreviations*: *CPAP* continuous positive airway pressure, *ETT* endotracheal tube, *FiO*_*2*_ fraction of inspired oxygen, *MAP* mean airway pressure, *PaO*_*2*_ partial pressure of arterial oxygen, *PCV* pressure-controlled ventilation, *PEEP* positive end-expiratory pressure, *PIP* peak inspiratory pressure, *PSV* pressure support ventilation, *RR* respiratory rate, *SD* standard deviation, *SIMV* synchronised intermittent mandatory ventilation

#Primary respiratory pathology = bronchiolitis and pneumonia, asthma, influenza, immersion injury

^Secondary respiratory pathology = airway management, sepsis, seizure management, tick paralysis, gastrointestinal bleeding, trauma, neurological injury, Guillain-Barre syndrome, ingestion and renal failure

*p < 0.05

In the CPT group, EELV changes at all measurement points were significantly greater (*p* < 0.001), indicative of either recruitment of atelectatic alveoli or further distention of already ventilated alveoli [6] (Table 2). The increase in EELV as a result of lung recruitment secondary to secretion removal is supported by the finding of movement of the geometric centre toward the dependent lung in the children receiving CPT (*p* = 0.005), indicating improved ventilation posteriorly. CPT mobilises secretions from peripheral airways of the lung where the secretions can cause collapse of distal alveoli, whereas suction removes secretions from the proximal airways and has minimal effect on peripheral secretion clearance. A higher global inhomogeneity index after CPT (*p* = 0.017) reflected greater variations in ventilation distribution and regionally opening lung fields.

**Table 2:** Difference between pooled routine airway clearance and chest physiotherapy data for each outcome measure, and the interaction effect of recruitment manoeuvres: mean difference, standard error (SE), significance and 95% confidence intervals (CI) (linear mixed models)

	Chest physiotherapy (CPT) minus routine airway clearance main effect				CPT*recruitment interaction effect
	Mean difference	SE	Significance	95% CI	Significance
**Ventilation distribution (relative impedance Δ)**					
Global Amp	− 0.004	0.012	0.745	− 0.027–0.020	0.479
Global EELV	0.084	0.018	*0.000	0.047–0.121	0.293
Anterior EELV	0.047	0.015	*0.003	0.017–0.078	0.931
Posterior EELV	0.107	0.027	*0.000	0.053–0.160	0.402
Global inhomogeneity index	0.043	0.018	*0.017	0.008–0.078	0.230
Geometric centre (%)	− 3.613	1.241	*0.005	− 6.097 to − 1.129	0.833
**Gas exchange**					
PaO_2_ (mmHg)	− 7.861	6.186	0.209	− 20.243–4.521	0.217
PaCO_2_ (mmHg)	9.615	3.013	*0.002	3.610–15.620	0.110
PF ratio	− 56.663	32.220	0.084	− 121.210–7.885	0.250
FiO_2_	0.040	0.024	0.106	− 0.009–0.089	0.279
SpO_2_	− 0.175	0.790	0.825	− 1.763–1.412	0.095
SpO_2_/FiO_2_	− 33.565	21.584	0.126	− 76.832–9.703	0.195
**Physiological state**					
Respiratory rate (bpm)	5.886	3.190	0.070	− 0.495–12.267	*0.001
Heart rate (bpm)	4.869	7.021	0.491	− 9.202–18.940	*0.048

*Abbreviations*: *Amp* amplitude, *bpm* breaths/beat per minute, *CI* confidence interval, *EELV* end-expiratory level volume, *FiO*_*2*_ fraction of inspired oxygen, *HR* heart rate, *PaO*_*2*_ partial pressure of arterial oxygen, *PaCO*_*2*_ partial pressure of arterial carbon dioxide, *PF* PaO_2_/ FiO_2_, *SE* standard error, *SpO*_*2*_ oxygen saturation, *Δ* change

*p < 0.05

No differences for global amplitude (*p* = 0.74) between those receiving CPT and those who did not were found, which is not unexpected as all participants were fully volume-controlled ventilated.

Improvements in EELV, geometric centre and global inhomogeneity occurred within 30 min of receiving CPT (*p* < 0.01) suggesting that by facilitating secretion clearance, CPT can result in immediate changes in ventilation distribution, which are sustained for up to 120 min and identifiable using EIT.

We have shown that EIT can detect regional changes in lung function as a result of CPT in ventilated infants and children, making it a potential clinical tool to measure the effects of CPT and for focusing CPT to areas of concern.

## Data Availability

The datasets used and/or analysed during the current study are available from the corresponding author on reasonable request.

## Abbreviations

CPT: Chest physiotherapy
EELV: End-expiratory lung volume
EIT: Electrical impedance tomography
ETT: Endotracheal tube
PaCO_2_: Partial pressure of arterial carbon dioxide
